# PathBank: a comprehensive pathway database for model organisms

**DOI:** 10.1093/nar/gkz861

**Published:** 2019-10-11

**Authors:** David S Wishart, Carin Li, Ana Marcu, Hasan Badran, Allison Pon, Zachary Budinski, Jonas Patron, Debra Lipton, Xuan Cao, Eponine Oler, Krissa Li, Maïlys Paccoud, Chelsea Hong, An C Guo, Christopher Chan, William Wei, Miguel Ramirez-Gaona

**Affiliations:** 1 Department of Biological Sciences, University of Alberta, Edmonton, AB T6G 2E9, Canada; 2 Department of Computing Science, University of Alberta, Edmonton, AB T6G 2E8, Canada; 3 Department of Plant Breeding, Wageningen University and Research, 6708 PB Wageningen, Gelderland, Netherlands

## Abstract

PathBank (www.pathbank.org) is a new, comprehensive, visually rich pathway database containing more than 110 000 machine-readable pathways found in 10 model organisms (*Homo sapiens, Bos taurus, Rattus norvegicus, Mus musculus, Drosophila melanogaster, Caenorhabditis elegans, Arabidopsis thaliana, Saccharomyces cerevisiae, Escherichia coli* and *Pseudomonas aeruginosa*). PathBank aims to provide a pathway for every protein and a map for every metabolite. This resource is designed specifically to support pathway elucidation and pathway discovery in transcriptomics, proteomics, metabolomics and systems biology. It provides detailed, fully searchable, hyperlinked diagrams of metabolic, metabolite signaling, protein signaling, disease, drug and physiological pathways. All PathBank pathways include information on the relevant organs, organelles, subcellular compartments, cofactors, molecular locations, chemical structures and protein quaternary structures. Each small molecule is hyperlinked to the rich data contained in public chemical databases such as HMDB or DrugBank and each protein or enzyme complex is hyperlinked to UniProt. All PathBank pathways are accompanied with references and detailed descriptions which provide an overview of the pathway, condition or processes depicted in each diagram. Every PathBank pathway is downloadable in several machine-readable and image formats including BioPAX, SBML, PWML, SBGN, RXN, PNG and SVG. PathBank also supports community annotations and submissions through the web-based PathWhiz pathway illustrator. The vast majority of PathBank's pathways (>95%) are not found in any other public pathway database.

## INTRODUCTION

Pathway diagrams are the roadmaps for molecular biology. Just as roadmaps show the connections between villages, towns, and cities, pathway diagrams illustrate the connections between genes, proteins and metabolites. A well-illustrated pathway diagram provides biological context to complex molecular processes in an easily understood and highly visual manner. In this regard, pathway diagrams provide a remarkably useful means for scientists to share, integrate, interpret and visualize ‘omics data and ‘omics measurements. Even now, compilations of pathway diagrams (i.e. pathway databases) are among the most frequently used and oft-referenced resources in the entire field of bioinformatics. This is because pathway databases are widely called upon to interpret genomics, metagenomics, transcriptomics, proteomics and/or metabolomics data. Furthermore, they are frequently used to facilitate more complex analytical tasks such as cellular modeling (1), flux balance analysis (2), gene-set enrichment analysis (3), metabolite-set enrichment analysis (4) and over-representation analysis (5).

A wide variety of pathway databases exist, including both commercial and open-access resources. Some of the best-known online pathway databases are also among the oldest. These include KEGG (6) and the ‘Cyc’ databases (7), both of which emerged in the mid 1990s. Both of these resources are superb collections, providing extensive, easily searched, web-accessible pathways and annotations that are widely used by thousands of scientists each day. More recently, a number of other pathway databases have emerged such as Reactome (8) and Wikipathways (9), which cover pathways for a selected (small) number of model organisms. Additionally, Wikipathways employs a community annotation model and exploits a number of continuing advances in web visualization and interactivity to offer a somewhat richer user/contributor experience. There are also a variety of specialized pathway databases such as BioCarta (https://cgap.nci.nih.gov/Pathways/BioCarta_Pathways) (10), InnateDB (11), PharmGKB (12) and the Pathway Interaction Database (13), which either focus on specific pathway types, specific (single) organisms or provide a different, often visually more impressive way of exploring pathways than their comprehensive pathway database cousins.

In addition to these freely available or open-access pathway databases, there are also several well-known commercial pathway databases such as GeneGo (https://portal.genego.com/), Protein Lounge (http://www.proteinlounge.com/) or IPA (Ingenuity Pathway Analysis) (https://www.qiagenbioinformatics.com/products/ingenuity-pathway-analysis/). In particular, the Ingenuity Pathway Analysis system by Qiagen is perhaps the most popular and most extensive commercial resource in terms of pathway coverage and pathway analysis tools.

While there are many excellent features in each of the pathway databases just mentioned, there are also some notable limitations as well. For instance, even though KEGG and the Cyc databases are explicitly small molecule pathway databases, they do not provide many pathways covering lipid synthesis, metabolite signaling, small molecule hormone signaling or small molecule drug action. While the KEGG database does provide some protein signaling, developmental and disease process pathways, the CycDBs do not. Neither KEGG nor the CycDBs provide pathways relating to cellular responses, and KEGG does not provide information on cellular locations or biological context. Being the oldest of today's pathway databases, KEGG and the Cyc databases also use somewhat dated approaches to web-based pathway visualization. While the Reactome and Wikipathways databases do provide somewhat more modern web interfaces and cover many aspects of protein and cellular signaling, they are also much more limited in their coverage of metabolism than KEGG or the Cyc databases. Likewise, Reactome and Wikipathways largely ignore metabolite signaling, hormone signaling, drug action and many common disease pathways. Even with their modern pathway visualization tools, the Reactome and Wikipathway pathway diagrams still lack the visual appeal or biological context of the pathway diagrams found in other pathway databases such as BioCarta (10), DrugBank (14) or SMPDB (15).

Ideally what is needed is a pathway database that combines the comprehensive metabolic pathway coverage found in the KEGG or the Cyc databases, with the rich protein/cellular signaling pathway coverage found in Reactome, Wikipathways or IPA, and the visualization quality and biological context found in BioCarta or SMPDB—into a single, freely available resource. Herein we describe just such a database—called PathBank. PathBank is a comprehensive, visually rich pathway database containing more than 110 000 machine-readable pathways found in 10 model organisms (including humans, rodents, plants, insects and microbes). PathBank provides detailed, fully searchable, hyperlinked diagrams of metabolic, protein/metabolite signaling, disease, drug and physiological pathways. Its pathway diagrams include information on the relevant organs, organelles, subcellular compartments, locations and structures to provide important biological context. All molecular/protein images are hyperlinked to databases with rich descriptions and all pathways are viewable using a modern ‘Google Maps’-like or AJAX-based visual navigation system. All of PathBank's pathways have detailed descriptions and all of its images, image maps, descriptions and reactions are freely downloadable in several common data exchange formats. The entire database may be browsed, searched or annotated using a variety of easy-to-use tools. Likewise, community or crowd-sourced annotations and submissions to PathBank are supported through the web-based PathWhiz (16) pathway illustrator.

## PathBank DESCRIPTION AND CONTENT

PathBank (version 1.0) contains 110 234 richly annotated, fully colored, machine-readable pathways for 10 model organisms (*Homo sapiens, Bos taurus, Rattus norvegicus, Mus musculus, Drosophila melanogaster, Caenorhabditis elegans, Arabidopsis thaliana, Saccharomyces cerevisiae*, *Escherichia coli* and *Pseudomonas aeruginosa*). The database currently contains 78 488 different compounds (including metabolites, drugs and other xenobiotics), 8993 different proteins and describes more than 176 535 different reactions and interactions. PathBank pathways are divided into two broad categories or classes: (i) Metabolite/Compound pathways and (ii) Protein pathways. Metabolite/Compound pathways contain a majority (>50%) of small molecules compared to proteins as pathway entities. Protein pathways contain a vast majority of proteins (typically >80%) compared to metabolites as pathway entities. In the current version of PathBank there are 109 836 Metabolite/Compound pathways and 398 Protein pathways.

Within PathBank there are six subcategories of Metabolite/Compound pathways: (i) Metabolic (catabolism/anabolism); (ii) Physiological/Endocrinological (primarily involving metabolites); (iii) Metabolite Signaling; (iv) Drug Metabolism; (v) Drug Action; and (vi) Disease (primarily involving small molecule metabolism). In contrast to the Metabolite/Compound pathways there are 15 subcategories of Protein pathways: (i) Immunological; (ii) Cellular Response; (iii) Gene Regulatory; (iv) Growth Factor; (v) Cytokine Signaling; (vi) Protein/Peptide Hormone-Mediated; (vii) Neurological Signaling; (viii) Developmental Signaling; (ix) Kinase Signaling; (x) Apoptosis Signaling; (xi) Stress Activated Signaling; (xii) Pathogen-Activated Signaling; (xiii) Transport/Degradation; (xiv) Cytoskeletal Signaling; and (xv) Disease (primarily involving protein dysregulation).

All pathways have detailed text descriptions that provide summaries of the pathway or the processes being depicted. Averaging a length of 342 words, these original descriptions highlight relevant background information, biological context and key reactions. Similarly, all compounds in PathBank have detailed text descriptions averaging 305 words. Further, all compounds have manually curated abbreviations for use in the easier-to-read large font pathway visualizations. Most of these compound names, abbreviations and descriptions were obtained from our own locally maintained databases such as HMDB (17), DrugBank (14), YMDB (18) and others. All protein names and descriptions are obtained from, and linked to, UniProt. Links and citation data for more than 12 958 different references (textbooks and journals) used to help create PathBank's pathways are also provided.

PathBank supports a wide variety of searching and browsing functions, including pathway browsing/searching (by name), compound browsing/searching (by name), protein browsing/searching (by name), sequence searching, compound similarity searching, molecular weight searching and advanced (Boolean) text searching. Users may also search PathBank by using lists of compound names, protein names and/or gene names or their corresponding identifiers (HMDB ID, Affymetrix ID, GenBank ID, UniProt ID, etc.). All of the pathways, proteins, and metabolites in PathBank are hyperlinked to other resources and all of the pathways are navigable through a modern visualization interface modeled after Google Maps (https://www.google.com/maps). PathBank also has a variety of tools to facilitate the display of specific pathway components and to map their relative concentrations. Similarly, different types of pathway displays are possible to facilitate printing or the preparation of slides and/or images. All of PathBank's pathway images, image maps, descriptions and reactions are freely downloadable in several common machine readable or data exchange formats including BioPAX, SBML, PWML, SBGN, RXN, PNG and SVG.

PathBank was implemented using a Ruby on Rails (http://rubyonrails.org, version 4.2.0) web framework incorporating a MySQL relational database (https://www.mysql.com, version 5.1.50) to manage all of the pathway data, including entity relationships, external references, descriptions, visualization specifications and chemical structures. PathBank uses the model–view–controller architecture, in which internal data logic is separated from user input and data presentation. The raw information stored in the database is dynamically extracted and rendered into web pages by PathBank's HTML interface responder. PathBank is hosted on a Digital Ocean server equipped with 4 CPUs, 50 GB of disk space, 8 GB of RAM and an additional 200 GB storage space on an Amazon S3 storage facility.

PathBank offers a number of features not found in other pathway databases. These features are highlighted in Table 1. In terms of the number of pathways per organism, PathBank offers the largest and possibly the most comprehensive collection of any public database. In terms of the total number of pathways, it is second only to KEGG (which has 653 007 pathways from 6076 organisms compared to the 10 organisms in PathBank). As shown in Table 1, PathBank also appears to provide much more visual detail, more biological context and more pathway-related data (text descriptions) than almost any other pathway database. In terms of biological context and detail, PathBank pathways illustrate organs, tissues, cells and organelles as well as physiological consequences (especially for drug action pathways). In terms of pathway-related data, PathBank provides almost twice as much text for its pathway descriptions as any other database, significantly more references than most other databases as well as uniquely providing both protein and metabolite descriptions (see Table 1). PathBank is particularly unique in its collection of lipid synthesis, disease, drug action, drug metabolism and metabolite signaling pathways. A key objective of PathBank is to provide pathway resources for the metabolomics and lipidomics communities. Technology now exists to detect and quantify thousands of individual lipids. Some of these lipids are unique to specific diseases, conditions, tissues or organisms. However, pathways describing the biosynthesis or degradation of these unique lipids are generally lacking in all current pathway databases (except PathBank). Indeed, because of these lipid pathways (as well as PathBank's many disease and drug action pathways) we estimate that >95% of the pathways found in PathBank are not found in any other pathway database. For instance, if pathway name similarity mapping is used to compare database overlap, <3% of PathBank's pathways are found in KEGG, <2% are found in Reactome, <1% are found in Wikipathways and <1% are found in BioCarta. Alternately, if one uses the average number of pathways/organism in each database and if one assumes all pathways in other databases are mostly covered in PathBank (a reasonable assumption) then only 1% of PathBank's pathways are found in KEGG, 11% are found in Reactome, 1% are found in Wikipathways and 3% are found in BioCarta.

**Table 1.** A comparison between PathBank and other pathway databases

	PathBank	KEGG	Reactome	Wiki-Pathways	BioCarta
No. of organisms	10	6076	16	31	2
No. of pathways	110 234	653 007	20 882	2785	590
Ave. no. pathways/organism	1 1 023	107	1305	90	295
Protein pathways?	Yes	Yes	Yes	Yes	Yes
Metabolic pathways?	Yes	Yes	Yes	Yes	No
No. of pathways (Human)	48 701	335	2227	1,102	314
No. of pathways (*E*. *coli*)	1178	119	0	12	0
No. of pathways (*A*. *thaliana*)	3356	138	0	93	0
No. of pathways (Mouse)	12 651	331	1649	225	276
Hyperlinked proteins/genes	Yes	Yes	Yes	Yes	No
Hyperlinked metabolites	Yes	Yes	Yes	Yes	No
No. of disease pathways	20 692	91	411	118	5
No. of signaling pathways	64	65	718	211	39
No. of drug action pathways	404	75	46	39	0
Compound descriptions	Yes	No	No	No	No
Protein descriptions	Yes	No	No	No	No
Pathway summaries	Yes	Yes	Yes	Yes	No
Ave. word count per summary	342	68	179	143	0
Ave. ref. count per pathway	12	14	3	8	0
Rich visual display	Yes	No	Yes	No	Yes
Displays protein 4° structure	Yes	No	Yes	No	No
Displays metabolite structure	Yes	Yes	Yes	No	No
Displays cellular organelles	Yes	No	Yes	Yes	Yes
Displays cellular locations	Yes	No	Yes	Yes	Yes
Displays organs/tissues	Yes	No	Yes (in EHLDs)	No	No
Multicolored pathways	Yes	No	Yes	Yes	Yes
Multiple pathway views	Yes	No	No	No	No
No. pathway image formats	2	1	6	7	1
XML compatible	Yes	KGML	Yes	Yes	No
SBML compatible	Yes	No	Yes	Yes	No
BioPAX compatible	Yes	No	Yes	Yes	No
‘Google Maps’ interactivity	Yes	No	Yes	No	No
Community editable	Yes	No	No	Yes	No
Open access/Free download	Yes	No	Yes	Yes	Yes
Integrated with other DBs	Yes	Yes	Yes	Yes	No
Integrated with other Apps	Yes	Yes	Yes	Yes	No

PathBank's collection consists of a large number of lipid synthesis pathways. A major goal of PathBank is to have a pathway for every protein and a map for every metabolite. Lipids constitute more than 70% of the nameable metabolite entities in most organisms but unfortunately there is a tendency for many biochemists and pathway databases to ignore lipids or to treat them as generic molecules. From the perspective of classical metabolism, the use of generic classes is helpful. However, from the perspective of metabolomics and lipidomics, this approach is very limiting. Indeed, the primary data outputs of lipidomics and metabolomics experiments are individual compound identities and concentrations for hundreds to thousands of unique (but often similar) molecules. Converting these data into ‘generics’ or compound classes leads to a significant loss of information. Furthermore, recent work in lipidomics is highlighting how important individual lipid molecules are for certain biological functions or disease processes (19–21). This is why there has been a strong push within the metabolomics and lipidomics community to develop a database like PathBank where the goal is to have ‘a pathway for every molecular entity’.

## PathBank LAYOUT AND NAVIGATION

**Figure 1.**
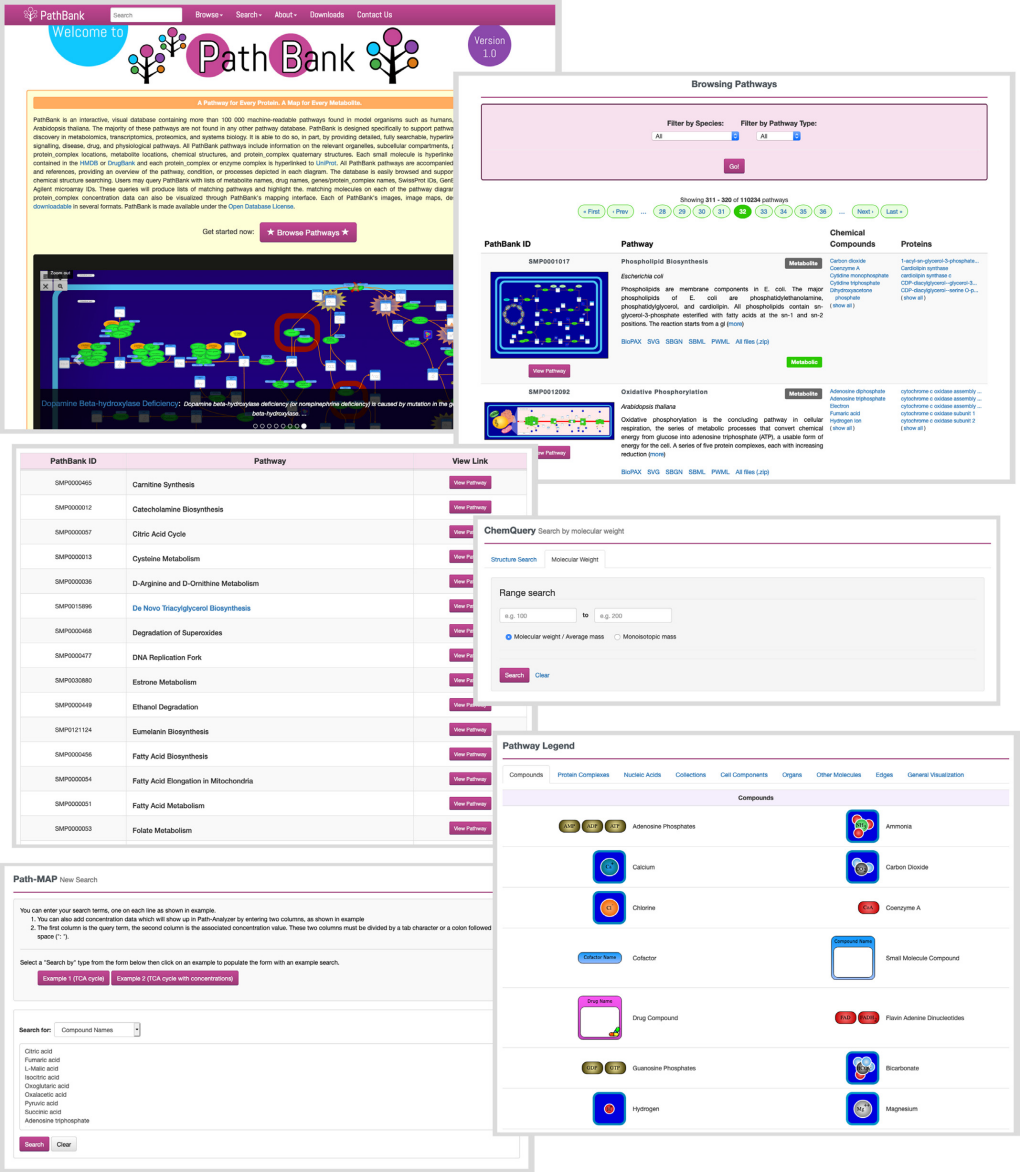
A screenshot montage of different browsing and searching screens taken from PathBank. A more detailed description of the different functions and capabilities of the various browse and search tools in PathBank is given in the text.

A screenshot montage of the PathBank interface and its search and browsing tools is shown in Figure 1. PathBank has a landing/home page very similar to that of its smaller cousin, SMPDB (15). This includes a short textual description of the database, a circulating ‘carousel’ of selected PathBank images and a series of tabs at the top of the page for navigation. An empty search box is located at the top left of each PathBank page. PathBank has five major tabs: (i) Browse; (ii) Search; (iii) About; (iv) Downloads; and (v) Contact Us. Under the Browse tab users may browse the website using four different pages: a) Pathways; b) Table of Primary Pathways (TOPP); c) Compounds (metabolites or drugs); or d) Proteins. All pathway search and browse results are filterable by species (10 model species available) and by pathway type (Metabolite/Compound and Protein) using two dropdown filters. Additionally, selecting a pathway type will render visible a third dropdown menu to allow filtering of the results by pathway sub-categories (6 Metabolite/Compound pathway sub-categories and 15 Protein pathway sub-categories).

If users choose to browse by Pathway, a multi-page table is presented that contains four columns: (i) the PathBank identifier (with the thumbnail image); (ii) the pathway details, which includes the pathway name, species, description and download links; (iii) the compounds (which are hyperlinked); and (iv) the proteins (which are also hyperlinked). Clicking on the thumbnail image of a given pathway will direct users to the full-size pathway image. Likewise, clicking on the metabolite/compound names (column iii) or protein names (column iv) will open a page with detailed compound descriptions (from the appropriate organism-specific metabolome database such as HMDB or from a specialized organism-specific database) or protein descriptions (from UniProt).

If the TOPP option is selected, a multi-page table is presented showing three columns: (i) the PathBank identifier; (ii) the pathway name including the species name (if filtered by all species); and (iii) the link to the pathway view. In the TOPP, similarly named (replicated) pathways are grouped together to facilitate browsing of largely unique pathways. Groupings are represented by a single primary pathway, hence the table name, which has a clickable pathway name displayed in blue text. These hyperlinked pathways may be selected by the user to navigate to a popover view containing all of the related pathways which can be similarly browsed. In the case of lipid pathways, only the generic version of the pathway is listed in the main table; the pathways for the specific lipids are browsable in the generic lipid's popover view.

Users can browse PathBank's compounds and proteins in much the same manner. Multi-page tables present compounds and proteins alphabetically in three columns: (i) the compound/protein's identifier along with the corresponding View buttons; (ii) the compound/protein's name (with a short description); and (iii) the associated pathways (which are hyperlinked). Clicking on a compound's View button will take a user to the appropriate ‘Compound Card’, corresponding to the database most appropriate for the chosen (or filtered) organism. The default is HMDB if no organism has been selected. Clicking on a protein's corresponding View button will take a user to the appropriate ‘UniProt Card’. Unlike the pathway browse results, compounds and proteins are filterable only by species using the dropdown menu provided. An additional filter in the form of a Search box at the top right of the table allows users to filter the page by compound, protein or pathway name.

PathBank places a strong emphasis on providing users with high quality, artistically pleasing pathway diagrams that are not only correct and informative but also colorful, interactive and richly detailed. All of the pathway diagrams in PathBank use scalable vector graphics (SVG) and a web interface technology inspired by Google Maps. This allows rapid and continuous zooming using a mouse pad or a mouse scroll wheel or through simply clicking on-screen zoom icons. It also allows facile navigation around zoomed-in pathway diagrams through a simple click-and-drag operation or through clicking on-screen up/down or left/right arrows located near the on-screen zoom functions. A full-screen view of each PathBank pathway diagram is also available, which can be toggled off and on by clicking the full-screen icon located between the navigation arrows.

On the right side of each pathway diagram is a pathway display panel with five tabs (Description, Highlight, Analyze, Downloads, Settings/Display). The default view is the Description panel, which describes the pathway in detail and provides one or more literature references. The Highlight panel (viewed by clicking the ‘Highlight’ tab) allows users to select and color different proteins and metabolites for display purposes. The Analyze panel (viewed by clicking the ‘Analyze’ tab) allows users to enter concentration (relative or absolute) data on proteins/transcripts and/or metabolites and to have these colored on the pathway diagram. It is highly recommended that users switch to the black and white version of the pathway when using the highlight and analyze tools in order to clearly see the elements being colored. The download panel (viewable by clicking the ‘Download’ tab) allows users to select the type of file format that they wish to have their pathway image (annotated or untouched) saved as. Options are available for downloading images in BioPAX (22) image format (full color SVG, grayscale SVG, simple SVG, large font SVG, and simple large font SVG), BioPAX only format, SBGN (23), SBML (24), PWML (a custom PathWhiz format), PNG or all of the above. At last, the Display panel (viewed by clicking the ‘Display’ tab) allows users to change the pathway display to suit a particular taste or application. Users may toggle between simple (thick line) and complex (individual lipid) membranes; between a dark blue (aqueous) background or a white background; between the full color, highly detailed version and the simplified KEGG-like (black and white) representation; and finally between full-names of pathway components and their large font abbreviations. There is also an additional fifth option to render the simplified (KEGG-like) pathway representation using the easier-to-read large font abbreviations.

In addition to these browsing functions, PathBank also offers extensive search functions. These include a general text search (available at the top of every PathBank web page) as well as several, more specific, search functions listed under the ‘Search’ tab. The text search supports Boolean logic (AND, OR and NOT operations) along with field-specific searches covering compound names, protein names, compound/protein identifiers, pathway names, and pathway descriptions. Instructions for the text search are accessible using the Search dropdown list. There are four additional searches available: (i) the Path-MAP Advanced Search; (ii) the ChemQuery Structure Search; (iii) the Molecular Weight Search; and (iv) the Sequence Search. The Path-MAP search allows users to enter long lists of compound names, protein names, compound identifiers, protein identifiers or gene identifiers to search for organism-specific pathways enriched with these entities. The result is a list of pathways with enrichment scores calculated using the frequency of matches and the number of pathway entities as scaled using a hypergeometric function. It is also possible to enter similar lists with Path-MAP but with concentrations or relative concentrations, as might be obtained from a typical proteomics, transcriptomics or metabolomics experiment. The result of this type of search is a pathway annotated and colored with the corresponding metabolite/protein concentrations according to a yellow (low)-red (high) concentration gradient. Other searches such as the ChemQuery and Molecular Weight searches are intended for metabolite or compound searching against PathBank's chemical database of 78 271 compounds and are identical to those used by HMDB, SMPDB and many other databases. The Sequence search uses BLAST (25) to find sequence matches or sequence similarities to the protein sequences within PathBank's sequence database of 8973 proteins.

PathBank's ‘About’ tab provides additional information about PathBank, its associated release notes, the required citations, database statistics, the PathBank style guide, links to other Pathway Databases, as well as images (via a Pathway legend) for the different components (organelles, organs, tissues) seen in PathBank pathways. PathBank's ‘Download’ tab allows users to download pathways, metabolite names and protein names (in CSV files) as well as all of its pathways in BioPAX, SBGN, SBML and PWML format. A subset of PathBank's pathways (i.e. the primary pathways browsable through the TOPP) are also downloadable in SVG and PNG format. Due to the extremely large file size, users who wish to download all pathway images will need to submit a request using PathBank's Contact form. All of PathBank's sequences (both gene and protein) are available in FASTA text format, all of its chemical structures are available in SDF format and all of its reactions are available in RXN format.

## PathBank ASSEMBLY, QUALITY CONTROL AND CURATION

**Figure 2.**
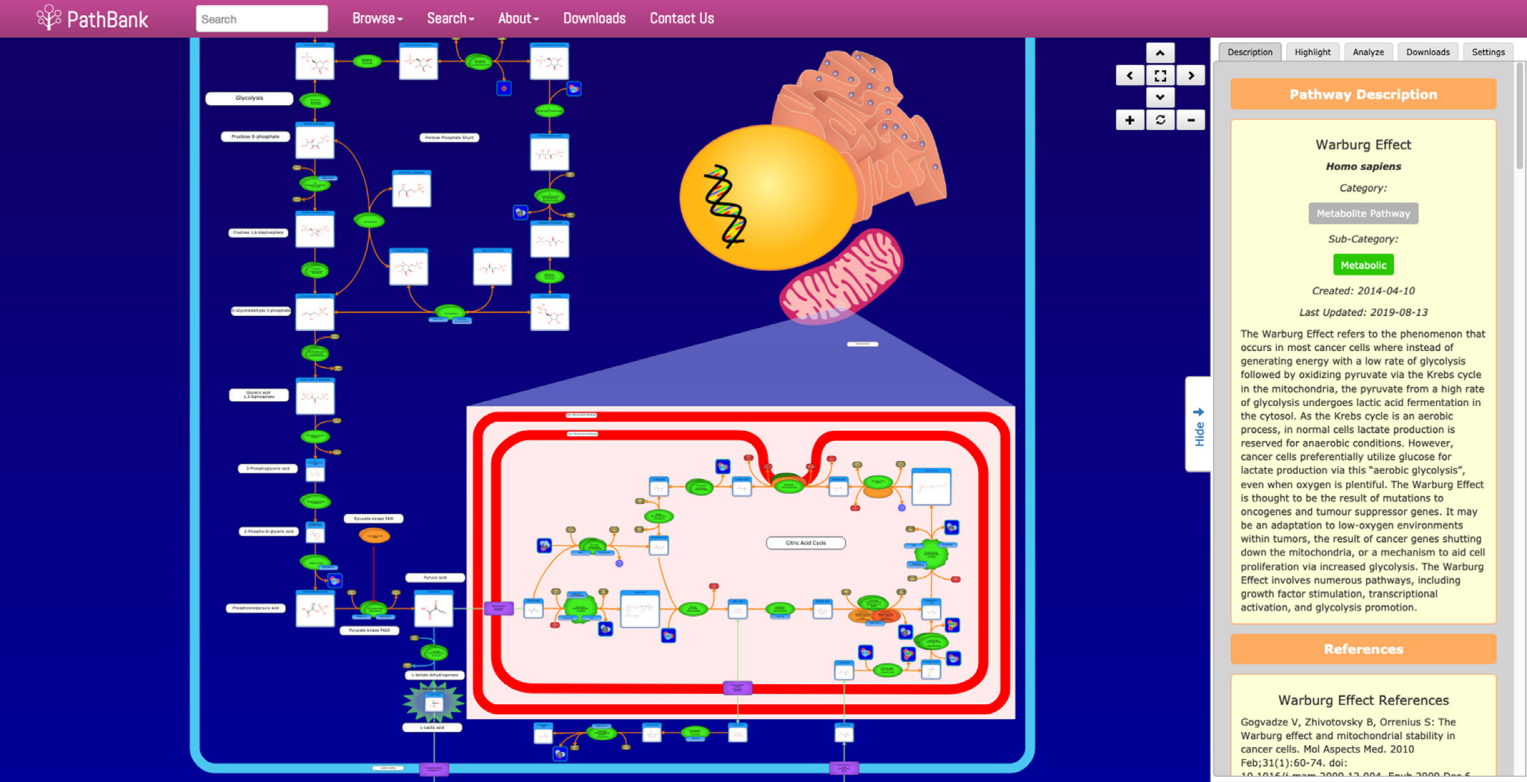
A screenshot from PathBank's ‘Warburg Effect’ pathway. First identified in the 1920’s, this is a pathway commonly activated in cancer and it is classified as a ‘Metabolite Disease’ pathway. Curiously, no other commonly used pathway database appears to illustrate this very well-known and important pathway.

PathBank was assembled using the PathWhiz pathway illustrator (16) using a team of pathway curators who have been manually researching, illustrating and refining pathway diagrams for more than 5 years. PathBank was initially built from a number of small molecule (metabolite) pathway diagrams that were originally assembled for SMPDB (15), HMDB (17), DrugBank (14), YMDB (18) and ECMDB (26). These pathway databases are restricted to depicting the metabolic or metabolite signaling pathways associated with humans (SMPDB, HMDB, DrugBank), yeast (YMDB) and *E. coli* (ECMDB). PathBank extends these existing pathway collections to cover other model organisms and to include disease, physiological, protein and cellular signaling pathways. The PathBank curation team took advantage of a number of recent improvements to the PathWhiz pathway illustrator (16,27). In particular, the latest version of PathWhiz has been enhanced to permit near-automated pathway propagation (to create homologous pathways for other species) and pathway replication (to create similar pathways within a species). PathWhiz has also been modified to support protein signaling, protein–protein interactions, protein–DNA interactions, transport and transcriptional events. An example of a PathBank pathway diagram generated via PathWhiz (illustrating the Warburg effect) is shown in Figure 2.

As described previously (15,16), all pathways constructed via PathWhiz are built according to standard operating protocols (SOPs), checklists and pathway illustration style guides to ensure general uniformity and consistency across pathways and across species. The comprehensive PathBank style guide, complete with numerous visual examples, is posted on the web page under the ‘About’ tab. In selecting which pathways would be drawn for a given species, members of the curation team would first perform an extensive literature search to identify unique metabolite classes or metabolic processes for the organism of interest. Second, a literature review would be done to search for unique proteins or unique protein/cellular signaling processes. Next, a detailed review of existing pathway diagrams (both metabolic and protein signaling) from multiple public pathway databases for that organism would be performed. These results would then be compiled and compared against existing internal reference pathway databases corresponding to the closest species (such as the HMDB for other mammals) to that organism. Further discussions and team meetings would be held wherein curators would discuss their findings with PathBank's lead curator (CL) and its principal investigator (DSW). Through this process new pathways for a given organism would be identified, selected (or rejected), assessed for their similarity to existing pathways, sketched (by hand) and finally manually drawn via PathWhiz (if unique) or replicated via PathWhiz and modified (if similar to another species) by members of the PathBank curation team.

After a given pathway was illustrated using PathWhiz, it would be evaluated independently by a second pathway curator (often the lead curator or the PI) to ensure it adhered to the PathWhiz style guides and SOPs. If the pathway failed to meet style guide requirements or failed to follow the SOPs, it would be corrected and re-evaluated until it met those requirements. Only after a pathway diagram passed secondary inspection would it be allowed to be propagated (to other species) or replicated (for related pathways within a species). This quality control protocol was applied to all hand-drawn primary pathways in PathBank which serve as template pathways that can be automatically replicated and/or propagated via PathWhiz. Spot checks (of ∼5% of the pathways) for any automatically replicated/propagated pathways were manually done by the lead curator(s) to ensure logical and biological consistency. Global or system-wide checks were also done periodically to ensure that organelle or organ/tissue images were appropriate for a given organism. Many of the primary pathways (especially for metabolism) that have been online for several years have undergone heavy remediations in order to comply with the guidelines outlined in PathBank's detailed style guide. As a result of this huge effort, these redrawn pathways have a standard, more aesthetically pleasing appearance, greater biological context (e.g. transporters across cell and organelle membranes) and increased connectivity between pathway entities. Many pathways have been updated with new descriptions and icons that have been drawn for PathBank's growing image collection. User feedback has also been used to further refine and improve these pathways.

All members of the PathBank curation team were required to have at least an undergraduate degree in bioinformatics or molecular biology. This ensured that they had sufficient biological and/or biochemical knowledge to understand the scientific literature and the biological processes being illustrated. All curation team members were also given extensive training by the lead curator(s) in pathway illustration via hands-on mentoring, text instructions, peer support, and video tutorials. However, it is important to note that given the inherently artistic nature of pathway illustration, the differences in artistic/illustrative skills among the curation team and the complexity of some biological processes, it is impossible to guarantee that all pathways in PathBank exhibit the same level of visual or conceptual quality.

Improvements and updates to PathBank's pathways are an ongoing process. Minor corrections or small improvements in image or pathway quality or pathway descriptions will be done without a formal update announcement. However, significant changes, additions or improvements to an individual pathway diagram (>10% in the number of pathway components), will be listed in the pathway description and the last updated date will be modified to reflect any such changes. As this is only version 1.0 of the PathBank database, all pathway diagrams are dated with August 2019 as the last update date. Large-scale updates and improvements to the database in the future will be given database version numbers (2.0, 3.0, etc.) and suitable database update dates. They will also be described in detail as publications or online update descriptions as appropriate.

PathBank is made available under the Open Database License in which users are free to contribute to the database using its PathWhiz pathway drawing tool. Indeed, one of the authors listed on this manuscript (MRG) contributed >50 primary pathways to PathBank as an external ‘community’ member. Of course, not all community contributions are of sufficient quality to merit inclusion to PathBank. Any decision to include/exclude community or crowd-sourced pathway submissions depends on the quality/quantity of the contribution and their adherence to PathBank's SOPs and style guide. The final decision for any external contribution is ultimately made by PathBank's lead curator. The PathBank curation team will closely work with, and will train, external pathway illustrators who are serious about contributing to this resource.

## PathBank LIMITATIONS AND FUTURE PLANS

Like all pathway databases, PathBank is a work in progress. Currently both the quantity and level of coverage of PathBank's protein pathways falls far short of what is currently available for PathBank's metabolite/compound pathways. Indeed, only 398 protein pathways have been illustrated to date, compared to 109 836 metabolite pathways. This imbalance reflects the past focus of the PathBank team's curation effort (which was primarily in metabolism), the extremely large number of lipid pathways (>90 000) and the challenges that exist in depicting the somewhat more complex protein signaling pathways. As PathBank's collection expands, so too will its genome coverage (currently ranging between 1.9 and 25.1%) for each model organism (viewable on PathBank's statistics page). Of course, even with this major annotation effort, not every known pathway will appear in PathBank—now or in the near future. Likewise, it is expected that a number of users will notice that some PathBank pathways do not depict all expected or relevant entities in the pathway diagrams. In these situations, we certainly encourage users to contact the PathBank curation team (via the ‘Contact’ tab) so that those corrections can be made or those PathBank depictions can be more fully explained.

Another clear limitation with PathBank is the relatively small number of model organisms that are currently covered. Currently, only 10 organisms are included in PathBank's ‘zoo’ with several well-known model organisms, including a few key vertebrates, several parasites and a number of important bacteria, not being included. Over the coming two years, it is expected that at least nine other model organisms will be added to the database, including the zebrafish (*Danio rerio*), the African clawed frog (*Xenopus laevis*), the chicken (*Gallus gallus*), rice (*Oryza sativa*), the malaria parasite (*Plasmodium falciparum*), the sleeping sickness parasite (*Trypanosoma brucei*) and several bacteria, including *Bacillus subtilis*, *Staphylococcus aureus*, and *Thermotoga maritime*. Additional organisms or species may be added depending on user feedback.

PathBank is also somewhat limited in the analytical tools that it offers. This limited offering was a deliberate decision. Because all of PathBank's data are freely downloadable, we believe it would be far better for PathBank to serve as a data resource hub rather than an analytical hub. By making PathBank more of a data resource hub, we hope to encourage bioinformatics specialists to download or use the PathBank data to develop and implement their own, much more sophisticated analytical tools and web servers. Indeed, this has already happened. For instance, PathBank's data is now being used to support a very popular analytical web server called MetaboAnalyst 4.0 (28).

PathBank was originally designed to facilitate interactive, web-based visualization and pathway navigation. However, this has led to the creation of pathways that are not particularly ‘printer friendly’. Indeed, many PathBank pathways have entity labels that are generally too small to be seen in conventional slides or manuscript figures. To improve the situation, PathBank now offers two alternative pathway displays (available through the Settings tab of the Display panel) that allows users to toggle between ‘regular text’ and ‘large text’ for both the full-color and simplified visualizations so that the image labels are more visible and printer-friendly. In order to accommodate the larger font size, manually curated abbreviations and gene symbols adhering to a strict seven-character limit are used in place of the longer full names of compounds and proteins. Additional efforts are ongoing to create even smaller or more compact pathway visualizations with less empty space and more grid-like entity layouts.

## CONCLUSION

PathBank represents a new type of pathway database. It has been designed to take some of the best features of earlier pathway databases and to combine them with newer, more modern kinds of web visualization tools and more advanced web interfaces to create a fully open access, visually rich and highly comprehensive pathway resource. PathBank also brings a number of unique features into the Pathway database field including exceptional pathway coverage, richly detailed pathway and entity descriptions, a number of new pathway types or categories (metabolite signaling, drug action, drug metabolism, disease-specific pathways), new kinds of pathway or entity visualization details (cellular location, organelles, organs, protein quaternary structure) and a much wider variety of pathway viewing and data exchange/storage options. Being the first release of this particular pathway database, we expect that users will note some missing elements or inconsistences with certain pathways or pathway data. However, we anticipate that through user feedback, continued community engagement and ongoing curation efforts, the next release of PathBank will be even more comprehensive and potentially much more useful to the community.
